# High Throughput SARS-CoV-2 Genome Sequencing from 384 Respiratory Samples Using the Illumina COVIDSeq Protocol

**DOI:** 10.3390/genes14030681

**Published:** 2023-03-09

**Authors:** Nasserdine Papa Mze, Idir Kacel, Mamadou Beye, Raphael Tola, Mariéma Sarr, Leonardo Basco, Hervé Bogreau, Philippe Colson, Pierre-Edouard Fournier

**Affiliations:** 1 UMR VITROME, Aix Marseille University, IRD, AP-HM, SSA, IHU—Méditerranée Infection, 13005 Marseille, France; 2 Service de Biologie, Unité de Microbiologie, Hôpital Mignot, Centre Hospitalier de Versailles, 177 rue de Versailles, 78150 Le Chesnay, France; 3 UMR MEPHI, Aix Marseille University, IRD, AP-HM, IHU—Méditerranée Infection, 13005 Marseille, France; 4 Unité de Parasitologie et Entomologie, Département de Microbiologie et Maladies Infectieuses, Institut de Recherche Biomédicale des Armées, 13005 Marseille, France

**Keywords:** SARS-CoV-2, MiSeq, Illumina, sequencing, clade

## Abstract

The emergence of the Coronavirus Disease 2019 (COVID-19) pandemic has fostered the use of high-throughput techniques to sequence the entire severe acute respiratory syndrome coronavirus 2 (SARS-CoV-2) genome and track its evolution. The present study proposes a rapid and relatively less expensive sequencing protocol for 384 samples by adapting the use of an Illumina NovaSeq library to an Illumina MiSeq flow cell instrument. The SARS-CoV-2 genome sequences obtained with Illumina NovaSeq and those obtained using MiSeq instruments were compared with the objective to validate the new, modified protocol. A total of 356 (94.6%) samples yielded interpretable sequences using the modified Illumina COVIDSeq protocol, with an average coverage of 91.6%. By comparison, 357 (94.9%) samples yielded interpretable sequences with the standard COVIDSeq protocol, with an average coverage of 95.6%. Our modified COVIDSeq protocol could save 14,155 euros per run and yield results from 384 samples in 53.5 h, compared to four times 55.5 h with the standard Illumina MiSeq protocol. The modified COVIDSeq protocol thus provides high quality results comparable to those obtained with the standard COVIDSeq protocol, four times faster, while saving money.

## 1. Introduction

The Coronavirus Disease 2019 (COVID-19) outbreak first emerged in Wuhan, Hubei Province, People’s Republic of China. It spread within a month to the rest of mainland China, then to neighboring countries, and finally to practically all countries in the world by January 2020. Since the end of the first quarter of 2020, all countries in the European Union have been affected by COVID-19 [1].

In France, the first imported cases of COVID-19 were detected on 24 January 2020 [2]. During the month of February 2020, different chains of transmission were discovered in several regions of the country. The main focus of contamination was the Oise region, situated to the north of Paris, the capital city of France. The spread of virus rapidly intensified by the end of February 2020 to reach an epidemic peak in the number of confirmed cases during the last week of March 2020. In the country, there were 38,453,595 confirmed cases of COVID-19 and 160,844 deaths, from 3 January 2020 to 10 February 2023, according to the World Health Organization (WHO) [3]. Currently in early 2023, the incidence and screening rates are decreasing in all age groups, especially among those under 10 years of age. The positivity rate is also decreasing in all age groups, except for those aged 70 years and older. In metropolitan France, the incidence, detection, and positivity rates are decreasing in all regions. New hospitalizations due to COVID-19 are also decreasing in most regions, stable in Pays de la Loire region, and increasing in Bretagne and Centre-Val de Loire regions at low levels. The number of deaths associated with COVID-19 is also continuing to decrease, except in Bretagne [4].

The COVID-19 pandemic, which has been on-going for more than three years since early 2020, has led to the widespread use of next-generation sequencing (NGS) of severe acute respiratory syndrome coronavirus 2 (SARS-CoV-2) genomes, which is essential to monitor the circulation of this virus and its evolution [5,6,7]. Numerous virus variants have emerged and have been identified successively, including B.1.177, B.1.160, α, β, γ, Delta, and, since late 2021, Omicron variants [8,9,10]. These variants have had different degrees of impact on public health, including increased human-to-human transmissibility, disease severity, and immune escape [11,12].

According to the French National Agency of Public Health (Santé publique France) of the Ministry of Health, as of October 2022, 651,751 SARS-CoV-2 genome sequences have been characterized and deposited in the national database by French laboratories since 2021 [13]. This NGS activity has been reinforced in France through the Emergen program launched in early 2021 by the French Ministry of Health and Ministry of Research. The main objective of the Emergen program is to follow the genetic evolution of the SARS-CoV-2 virus to detect the emergence of variants and their spatio-temporal distribution in the country. SARS-CoV-2 genomics has allowed the surveillance of the emergence and spread of new variants [14]. Indeed, the predominant SARS-CoV-2 variants in France were the B.1.160 Pangolin lineage (or Marseille-4 variant) between August 2020 and January 2021, the α variant since February 2021, the Delta variant since July 2021, and the Omicron variants since January 2022 [15]. At present (in early 2023), the predominant variant is BA.5.

At the University Hospital Institute-Méditerranée Infection, SARS-CoV-2 genomic surveillance began with the first diagnosis of SARS-CoV-2 infection, and variants were detected in the summer of 2020 using Oxford Nanopore MinION/GridION NGS and Illumina MiSeq then NovaSeq platforms. For the Oxford Nanopore platform, the GridIOn instrument is used to sequence multiple samples and track the results in real time. Using nanopore technology, a rapid sequencing protocol was developed to sequence the entire SARS-CoV2 genome in a single sample in less than two hours (first author, personal communication). However, nanopore technology is limited to sequencing a maximum of 96 samples. As for the Illumina platform, the NovaSeq instrument allows sequencing of up to 1536 samples in two flow cells of 2 lines each (i.e., 384 samples/line × 4 lines in two flow cells). By comparison, the Illumina MiSeq standard protocol is designed to sequence 96 samples simultaneously in a MiSeq flow cell.

Recently, however, it has been reported that the sequencing capacity of a modified Illumina MiSeq protocol can be increased from 96 to 384 samples in a single flow cell [6], allowing a greater throughput sequencing. In the present study, we developed a protocol that considerably increases the throughput capacity of Illumina MiSeq sequencing using a dedicated NovaSeq library for NovaSeq instrument. In a previous study conducted by Bhoyar et al. [5], it was shown that the NovaSeq library can be used to sequence 96 samples in a MiSeq flow cell. The objective of the present study was to adapt the NovaSeq library, originally designed to sequence 384 samples in the NovaSeq instrument, to sequence SARS-CoV-2 genomes from 384 samples using the MiSeq instrument in a single flow cell, thus maximizing the throughput sequencing capacity in a single run to 1536 samples (4 flow cells × 384 samples/flow cell), to compare the results obtained on the NovaSeq and MiSeq instruments.

## 2. Materials and Methods

### 2.1. Virus Samples

Nasopharyngeal swab specimens were collected from patients who presented spontaneously for routine diagnosis of SARS-CoV-2 at the University Hospital Institute-Méditerranée Infection, Marseille, southeastern France. Nasopharyngeal swabs were collected and sent to our laboratory for the diagnosis of SARS-CoV-2 infection by real-time reverse transcription-polymerase chain reaction (RT-PCR), as previously described [10]. Genome sequencing was performed on SARS-CoV-2 RNA-positive samples, as recommended by the French Ministry of Health [16]. All clinical samples were fully anonymized. The present study was reviewed and approved by the Ethics Committee of the University Hospital Institute-Méditerranée Infection under reference no. 2022-017.

### 2.2. RNA Extraction

Viral RNA extraction from nasopharyngeal swab specimens was performed based on the MVP_2Wash_200_Flex protocol using an automated nucleic acid purification system (Thermo Scientific KingFisher Flex Purification System; Woodlands, Singapore), following the recommendation of the manufacturer (cat. no. A42352). Extracted RNA was stored at −20 °C until use.

### 2.3. Standard Illumina CovidSeq Protocol

Libraries were prepared following the Illumina COVIDSeq protocol (Illumina Inc, San Diego, CA, USA). There were four steps in the protocol: cDNA preparation, target amplification, library preparation and pooling, and sequencing. To prepare cDNA (first step), 8.5 µL of extracted RNA were mixed with 8.5 µL of elution prime fragment 3HC mix to anneal RNA. The annealed RNA was transcribed into cDNA using 1 µL of reverse transcriptase HT. For target amplification, the synthesized cDNA was amplified (second step) by performing two multiplex PCRs with two non-overlapping primer pools (COVIDSeq Primer Pool 1 and COVIDSeq Primer Pool 2) comprising the ARTIC v3 SARS-CoV-2 specific primer set [17]. The PCR amplification conditions were as follows: initial denaturation at 98 °C for 3 min, followed by 35 cycles including denaturation at 98 °C for 30 s then annealing at 65 °C for 5 min. Amplicons were tagged onto 4 µL Enrichment BLT HT beads for library preparation (third step). The PCR-amplified product was processed for tagging and adapter ligation with 10 µL of the Illumina IDT^®^ PCR Indexes Sets 1–4 (384 indexes, for 384 samples). The labeled amplicons were amplified by PCR under the following conditions: initialization at 72 °C for 3 min, initial denaturation at 98 °C for 3 min, followed by 7 cycles with 20 s at 98 °C for, 30 s at 60 °C, 1 min at 72 °C, and a final extension step at 72 °C for 3 min. PCR products were batch processed in four 96-well plates. COVIDSeq HT (CPC HT) positive control and a negative control were placed in each 96-well plate. A defined volume (5 µL) of all 96 amplicons was pooled in a 1.5 mL microfuge tube for each plate and quantified using the Qubit dsDNA HS assay kit on a Qubit fluorometer (Invitrogen, Villebon sur Yvette, France). Each pool was diluted 1:10 with resuspension buffer, and the concentration was adjusted to 4 nM as recommended by Illumina. To perform sequencing (fourth step), 25 µL of each pool at 4 nM were pooled in a 1.5 mL microfuge tube to constitute the final library. The library (2.25 µL) was diluted to a final concentration of 0.5 nM, denatured, and neutralized with 4 µL of 0.2 N NaOH and 5 µL of 400 mM Tris-HCl. The denatured library was mixed with 63 µL of ExAmp mix and placed in the SP flow cell according to the NovaSeq-XP workflow (Illumina Inc). The reads length obtained was 50 bp.

### 2.4. Modified Illumina COVIDSeq Protocol

To sequence 384 samples, the final library that was obtained from four pools at 4 nM, as described above in the standard CovidSeq Illumina protocol, was used. cDNA samples (7 µL of the library) were denatured by adding 7 µL of 0.2 N NaOH and incubated for 5 min. A total of 986 µL of neutralizing buffer (HT1) were added to obtain the final volume of 1000 µL of library. The final library (180 µL) was added to HT1 (420 µL). PhiX (6 µL, 20 pM) was added to the latter mixture by replacing 6 µL of the library-HT1 mixture with 6 µL of PhiX. The library-HT1-PhiX mixture was placed in MiSeq sequencing cartridge at a final concentration of 24 pM to adjust the density to 1347 k/mm^2^ which avoids overclustering of the flow cell (MiSeq reagent kit V2, Illumina Inc.). Sequencing was performed as recommended in the MiSeq sequencing guide (Illumina Inc.) and the reads length obtained was 250 bp.

Before sequencing 384 samples, we first sequenced 96 samples, as recommended by Bhoyar et al. [5], to obtain a cluster density of 1005 k/mm^2^, a passing filter of 88.3%, and Q30 quality scores of 81.6%. We sequenced 192 samples to obtain a cluster density of 1013 k/mm^2^, a passing filter of 91.5%, and Q30 quality scores of 81.5%. Finally, 384 amplicons were sequenced to obtain a cluster density of 1347 k/mm^2^, a passing filter of 81.4%, and Q30 quality scores of 76.5% (Table 1).

### 2.5. NGS Data Analysis

#### 2.5.1. Standard Illumina COVIDSeq Sequencing Data

Sequence read processing and genome analysis were performed as previously described [18,19]. Briefly, base calling was carried out using the Dragen Bcl Convert pipeline v3.9.3. The bwa-mem2 tool v2.2.1 was used for mapping, which was based on Wuhan-Hu-1 isolate genome (GenBank accession no. NC_045512.2) [20]. The resulting sequence was cleaned using SAMtools program V1.13 [21,22]. FreeBayes v1.3.5 was used for variant calling [23,24]. Consensus genomes were built with the Bcftools program v1.13 [25]. All nucleotide and deduced amino acid sequences were compared to Wuhan-Hu-1 isolate genome, and sequence modifications were detected using Nextclade tool [26,27,28]. Nextstrain clades and Phylogenetic Assignment of Named Global Outbreak Lineages (PANGOLIN) were identified with the Nextclade web application [26,27,28] and Pangolin tool [29,30], respectively.

#### 2.5.2. Modified Illumina MiSeq Sequencing Data

Sequence reading and analysis were performed as described in our previous work [4]. Trimmomatic v0.39 was used to trim raw reads and ensure quality control of sequences [31]. The alignment of the trimmed reads to the reference SARS-CoV-2 genome was performed using minimap2 (v2.17 r941) [32]. Using SAMtools (v. 1.13), the sam files of the mapping were sorted and converted into bam files [22]. A consensus sequence of the genomes in fasta format was generated using sam2consensus [33]. Sample parameters and metrics that assess the quality of the consensus genomes are shown in Supplementary Table S1.

### 2.6. Statistical Analysis

The mean values were compared using the two-sided Student *t*-test. The significance level was set at *p* < 0.05. All statistical tests were performed using Excel (Microsoft, Redmond, WA, USA).

## 3. Results

Of 376 samples processed for sequencing with the modified Illumina COVIDSeq protocol, 356 (94.7%) yielded interpretable genome sequences as defined by Nextclade (https://clades.nextstrain.org/, accessed on 2 September 2022). Of these, 280 (78.7%) yielded high-quality sequences with at least 90% coverage of the SARS-CoV-2 genome and an average sequence depth (± standard deviation [SD]) of 509.6 ± 120.94 x (range, 160.0–948.1). Seventy-six samples had a coverage between 38.5% and 89.8% and a mean (±SD) depth of 216 ± 119 x (range, 35.5–638.8) (Supplementary Table S2). The MiSeq genome sequences with a good quality (*n* = 329) were submitted to GenBank [34] (accession numbers OP606806–OP607134).

Sequencing failed in 20 samples for which viral load was medium or low as assessed by qPCR cycle threshold values (CT) ranging from 23.0 to 33.7 cycles. For all samples from which SARS-CoV-2 genomes were sequenced with the modified COVIDSeq protocol, an average of 41.4 mutations were found. In addition, 35 different SARS-CoV-2 lineages were detected among the 356 samples that yielded interpretable genome sequences with the modified COVIDSeq protocol.

Of 376 samples sequenced using the standard Illumina COVIDSeq protocol, 357 (94.9%) yielded interpretable genome sequences as defined by Nextclade. Of these, 305 (85.4%) yielded high-quality sequences with at least 90% coverage of the SARS-CoV-2 genome and an average depth (±SD) of 2988.0 ± 563.5 x (range, 795.0–4393.0). Fifty-two samples had a coverage between 45.5% and 89.5% and a mean (±SD) depth of 2627.3 ± 616.7 x (range, 1187.0–4032.0) (Supplementary Table S2). Sequencing failed in 19 samples (5.1%). Sequencing also failed with these 19 samples when analyzed with MiSeq instrument. Among samples sequenced using Illumina COVIDSeq standard protocol, an average of 39.6 mutations were found. A total of 34 distinct SARS-CoV-2 lineages were detected among 357 samples that yielded interpretable sequences using the standard COVIDSeq protocol. NovaSeq genome sequences with a good quality (*n* = 344) were submitted to GenBank (accession numbers OP606462–OP606805).

All 356 samples from which interpretable genome sequences were obtained using the modified COVIDSeq protocol were also successfully sequenced with the standard COVIDSeq protocol. Only one sample (0.3%) yielded an interpretable genome sequence (GenBank no. IHUCOVID-057737-Nova1M/2021) with the standard COVIDSeq protocol but not with the modified COVIDSeq protocol. However, the genome coverage for this sample was 49.3%, and the sequencing depth was 3045x.

For samples with SARS-CoV-2 genome sequenced successfully with both methods (*n* = 356), PANGOLIN assigned the same lineage in 343 of 356 variants (96.9%) (Supplementary Table S3) and indicated a dominant occurrence of BA.1.17 (*n* = 69) and BA.1.18 (*n* = 43) lineages. In two samples, MiSeq consensus genome was assigned to the AY.122 variant, while the COVIDSeq consensus genome was assigned to the AY.122.6 lineage of this variant. For an additional sample, the MiSeq consensus genome was assigned to the AY.34 variant, while the COVIDSeq consensus genome was assigned to the AY.34.1 lineage. Also, there were six samples for which the MiSeq consensus genome was assigned to the BA.1 variant, while the COVIDSeq consensus genome was assigned to the BA.1.1 lineage. Sequencing depth was higher for sequences obtained using the COVIDSeq protocol (mean ± SD, 2935.7 ± 584.7) compared to those obtained with the modified COVIDSeq protocol (446.8 ± 170.3), and the difference was significant (*p* = 0.0001). Coverage was similar between the two methods (mean ± SD, 94.6 ± 9.7% versus 91.6 ± 11.9% for the COVIDSeq protocol and the modified protocol, respectively; *p* = 0.84). There were more mutations detected (41.3 versus 37.4 on average) with the modified COVIDSeq protocol, but the difference was not significant (*p* = 0.74). Otherwise, both protocols assigned the variants to the same clades based on the nomenclature defined by Nextstrain, suggesting that a majority (i.e., 279 of 356 [78.4%] genomes) of the samples belonged to clade 21K while 65 (18.2%) belonged to clade 21J. Four variants belonged to clade 21I, two to clade 20A, one to clade 21L, and five to clade 20I (Table 2). 

**Figure 1:**
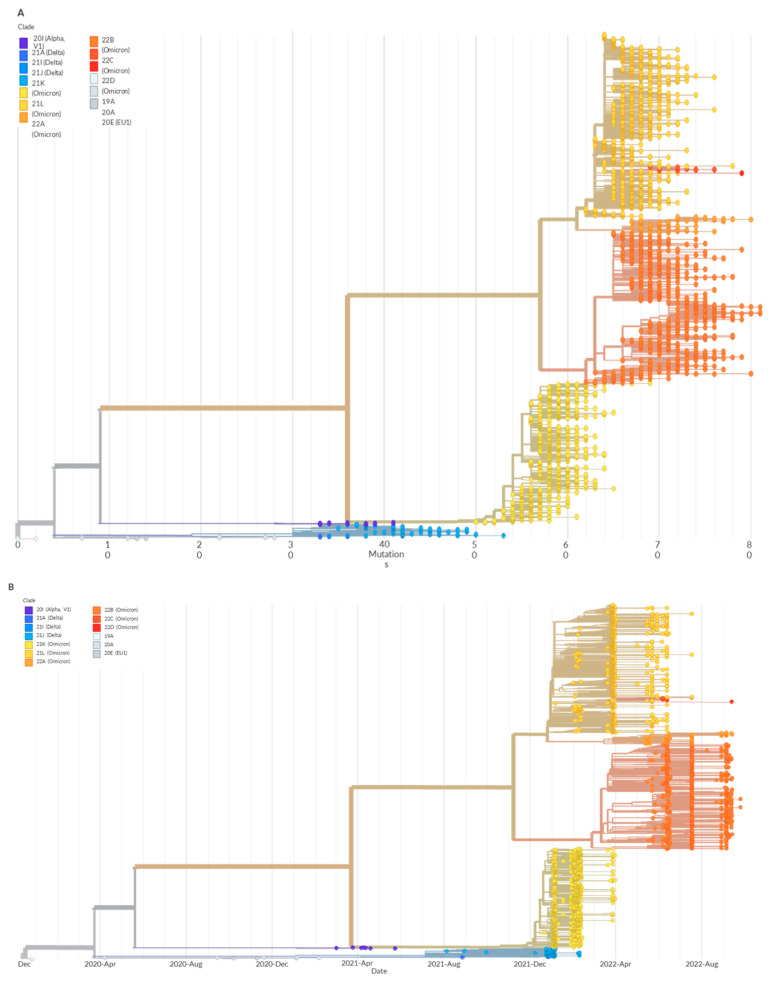
Phylogenetic distribution of SARS-CoV-2 genomes. (**A**) Phylogenetic trees generated by Nextstrain [29]. Of 3384 genomes, 2519 are highlighted. Clade 22 B is the dominant clade. This phylogenetic analysis shows different variants present in all sequences and their mutational profiles. (**B**) Phylogenetic trees showing the evolution of SARS-CoV-2 variant strains.

Therefore, our results strongly suggest that high quality genomic sequences can be obtained by multiplexing 384 NovaSeq libraries in a single MiSeq flow cell. This methodological approach allowed us to genotype 3384 samples to detect mutation patterns, identify SARS-CoV-2 lineages, and follow their chronological evolution (Figure 1A,B).

Regarding the financial cost of the two protocols, it was shown that the estimated cost to sequence 384 samples was approximately 6794 euros using the modified COVIDSeq protocol, as compared to 8025 euros using the standard COVIDSeq protocol (Table 3). Therefore, the standard COVIDSeq protocol was more expensive. Regarding the time of run (without including sequence analysis time), it was 22 h with the standard COVIDSeq protocol, as compared to 52 h with the modified COVIDSeq protocol.

## 4. Discussion

Researchers worldwide are being encouraged by international organizations and governments to collect and sequence SARS-CoV-2 genomes and share sequence data through platforms like GenBank or Global Initiative on Sharing Avian Influence Data (GISAID) where currently (as of February 2023) more than 14,919,562 genome sequences from many countries are available [35]. Databases with sequences from different periods of time and laboratories around the world would facilitate the monitoring of SARS-CoV-2 and the understanding of its past, current, and potential evolutionary changes. Therefore, the availability of relatively simple, rapid, high throughput and the cheapest possible sequencing methods is of great interest.

Recently, a new protocol for sequencing SARS-CoV-2 genomes using a COVIDSeq library was described [5]. Such a library is usually used to perform NGS on a NovaSeq instrument that is very expensive (about one million euros). The optimized protocol used a COVIDSeq library on a MiSeq instrument that is far less expensive (approximately four times) than the NovaSeq instrument. However, in the study conducted by Bhoyar et al. [5], only NGS was performed for 96 samples in a flow cell, which means that it would take a much longer time and would need four flow cells to sequence 384 amplicons. In the present study, we developed and validated a simple, fast, and cheaper method to sequence 384 SARS-CoV-2 genomes in a single run, in a single flow cell, on a MiSeq instrument. Minor modifications to the protocol developed by Bhoyar et al. [5] were necessary to achieve the NGS of 384 samples in a single flow cell.

The modified Illumina COVIDSeq protocol provided high quality sequences in our experience despite the increased number of samples to be processed simultaneously. A greater sequencing depth was obtained with the standard COVIDSeq protocol than with our modified COVIDSeq protocol, which might be the result of the fact that the performance of the NovaSeq instrument produces a large amount of high-quality sequences. Nevertheless, the final genome sequences of various SARS-CoV-2 lineages obtained using both protocols were identical for all. Interestingly, at lower, but still substantial sequencing depth, more mutations were identified using the modified COVIDSeq protocol. 

In the present study, we have shown that it is possible to sequence amplicons obtained from 384 SARS-CoV-2-positive samples in a single flow cell by slightly modifying the standard COVIDSeq protocol. Our modified protocol allowed to sequence SARS-CoV-2 genomes from 3384 samples, contributing to the monitoring of the circulation and evolution of SARS-CoV-2 variants in southern France. As a matter of fact, in practice, this modified COVIDSeq protocol was used as a backup method when our NovaSeq instrument was not operational. Hence, this strategy contributed efficiently to local and national SARS-CoV-2 genomic surveillance. Furthermore, if we compare the cost to perform the modified COVIDSeq protocol for NGS on the MiSeq instrument to the one that our team previously also reported for NGS on the MiSeq instrument [4], the total cost to sequence 384 samples was estimated to be 6794 euros with the modified COVIDSeq protocol compared to 15,380 euros with the modified MiSeq protocol. Therefore, based on our cost–benefit estimation, the modified COVIDSeq protocol saves 9603 euros per 384 samples. This difference is largely due to the fact that Illumina COVIDSeq reagent is less expensive than the MiSeq reagent, especially when purchased to sequence large numbers of samples (e.g., 33,700 euros for 3072 samples). Moreover, it takes 52 h (without the time required for sequence analysis) to sequence 384 samples regardless of the protocol (modified MiSeq or modified COVIDSeq protocols). If we compare the standard MiSeq protocol, the cost of which has been estimated to be 21,000 euros for 55.5 h [4], to the modified CovidSeq protocol, the latter saves approximately 15,223 euros and 2 h.

## 5. Conclusions

The standard COVIDSeq Illumina protocol can be modified to sequence 384 amplicons simultaneously in a single flow cell without compromising the high-quality sequences. This modified protocol is less expensive, more rapid, and compatible with the Illumina MiSeq sequencer. This approach may be a promising SARS-CoV-2 genome NGS strategy, notably in a situation when a NovaSeq instrument is not available or operational. This modified protocol could be used in countries that only have the Miseq sequencer, especially in low-income countries, which could allow them to conduct surveillance for SARS-CoV-2 variants at a lower cost.

## Figures and Tables

**Table 1:** Different protocols of library preparation to sequence amplicons using modified protocols.

Methods	Number of Amplicons Tested	Volume of Library (µL)	Cluster Density (K/mm²)	Passing Filter (%)	Estimated Yield (Mb)	Q30 (G/%)
Bhoyar et al. 2021 [5]	96	5	unknown	unknown	unknown	unknown
COVIDSeq modified protocol	96	5	1005	88.3	8862	6.9/81.6
	192	7	1013	92.0	9274	7.2/81.5
	384	7	1347	81.4	10,244	7.5/76.5

COVIDSeq modified protocol is the method used in this study.

**Table 2:** Comparison of SARS-CoV-2 clades monitored with two sequencing protocols.

Technique	20A	20I	21I	21J	21K	21L	Total
MiSeq	2	5	4	65	279	1	356
NovaSeq	2	5	4	66	279	1	357

**Table 3:** Comparison of financial costs and required estimated time for two sequencing protocols.

Technique	Reagent	Manufacturer	Total Duration (h)	Cost (euros)	Total Cost (euros)	
					NovaSeq	MiSeq
protocol modified for COVIDSeq and MiSeq (for preparation of library)	Illumina COVIDSeq™ Test (384 Samples)	Illumina	8	4212	8025	6794
	IDT for Illumina PCR Indexes Set 1–4	Illumina		1267		
	Illumina COVIDSeq v4 Primer Pools, 384 Samples RUO	Illumina		235		
	Qubit dsDNA HS Assay Kit	Thermo Fisher		60		
NovaSeq	NovaSeq 6000 SP Reagent Kit (100 cycles) V1.5	Illumina	14	1971		
	NovaSeq XP 2-Lane Kit V1.5	Illumina		280		
MiSeq	MiSeq Reagent Kit V2 (500 cycles)	Illumina	44	1020		

## Data Availability

The high-quality genomic sequences were submitted to the GenBank (accession numbers OP606806-OP607134 for the modified Illumina COVIDSeq protocol and OP606462-OP606805 for the Illumina COVIDSeq protocol).

## Supplementary Materials

The following supporting information can be downloaded at: https://www.mdpi.com/article/10.3390/genes14030681/s1, Table S1: Data generated from the modified Illumina COVIDSeq protocol; Table S2: Data generated from the Illumina COVIDSeq procedure; Table S3: Data generated for comparison between data from the modified Illumina COVIDSeq protocol and from the Illumina COVIDSeq procedure.

## Abbreviations

COVID-19: coronavirus disease 2019; C_T_, cycle threshold; GISAID, Global Initiative on Sharing Avian Influence Data; Mbp, megabase; NGS, next generation sequencing; PANGOLIN, Phylogenetic Assignment of Named Global Outbreak Lineages; pb, base pair; RSB, Resuspension Buffer; RT-PCR, reverse transcription-polymerase chain reaction; SARS-CoV-2, severe acute respiratory syndrome coronavirus 2; SD, standard deviation; WHO, World Health Organization.
